# Towards Lipidomics of Low-Abundant Species for Exploring Tumor Heterogeneity Guided by High-Resolution Mass Spectrometry Imaging

**DOI:** 10.3390/ijms141224560

**Published:** 2013-12-17

**Authors:** Jonathan Cimino, David Calligaris, Johann Far, Delphine Debois, Silvia Blacher, Nor Eddine Sounni, Agnès Noel, Edwin De Pauw

**Affiliations:** 1 Mass Spectrometry Laboratory, GIGA-R, Department of Chemistry, University of Liege, Liege 4000, Belgium; E-Mails: j.cimino@ulg.ac.be (J.C.); johann.far@ulg.ac.be (J.F.); delphine.debois@ulg.ac.be (D.D.); 2 Laboratory of Tumor and Development Biology, GIGA-Cancer, University of Liege, Liege 4000, Belgium; E-Mails: silvia.blacher@ulg.ac.be (S.B.); nesounni@ulg.ac.be (N.E.S.); agnes.noel@ulg.ac.be (A.N.); 3 Department of Neurosurgery, Brigham and Women’s Hospital, Harvard Medical School, Boston, MA 02115, USA

**Keywords:** MALDI mass spectrometry imaging, FTICR mass spectrometry, lipidomics, hypoxia, necrosis, cancer

## Abstract

Many studies have evidenced the main role of lipids in physiological and also pathological processes such as cancer, diabetes or neurodegenerative diseases. The identification and the *in situ* localization of specific low-abundant lipid species involved in cancer biology are still challenging for both fundamental studies and lipid marker discovery. In this paper, we report the identification and the localization of specific isobaric minor phospholipids in human breast cancer xenografts by FTICR MALDI imaging supported by histochemistry. These potential candidates can be further confirmed by liquid chromatography coupled with electrospray mass spectrometry (LC-ESI-MS) after extraction from the region of interest defined by MALDI imaging. Finally, this study highlights the importance of characterizing the heterogeneous distribution of low-abundant lipid species, relevant in complex histological samples for biological purposes.

## 1. Introduction

The lipid family is composed of a wide range of molecular species that include triacylglycerides, sphingolipids, phosphoglycerides and sterols [1]. They are involved in physiological processes regulated by thousands of metabolic pathways (e.g., fusion/division [2,3] or apoptosis [4], cell shape [3], membrane vesicle trafficking [5], signal transduction [6]). Alterations of these pathways are associated with a variety of human diseases such as cancer [7], neurodegenerative diseases [8,9], heart disease [10,11] or diabetes [12].

As for genomics and proteomics that aim at studying genes and proteins respectively, tools have been developed in the field of lipidomics for a comprehensive exploration of lipids [13]. The detection and localization of biomolecules are generally performed by chemical imaging techniques such as dye staining and immunohistochemistry. However, common lipid stainings which target neutral lipids [14–17] are limited to the study of a few lipids in tissues. Moreover, when immunohistochemical methods are employed, the use of antibodies raised against different phospholipid head groups does not allow the individual localization of specific lipid molecular species in tissues. Consequently, a powerful method for the direct localization and characterization of lipids would bring a significant advance to the field. To identify lipids, spectroscopic methods including nuclear magnetic resonance spectroscopy, fluorescence spectroscopy, as well as mass spectrometry (MS) have been used and coupled with separation methods [18,19]. However, this requires the preliminary extraction of the lipids and result in the loss of localization information. Improvements in mass spectrometry have led to an unprecedented capacity to characterize lipid species directly on tissue sections or after extraction. For instance, matrix-assisted laser desorption/ionization mass spectrometry imaging (MALDI MSI) is an emerging application for lipid research and has been applied for the *in situ* identification and localization of membrane lipids on tissue sections of lung [20], brain [21] and kidney [22] without the requirement of specific probes or staining. Liquid chromatography (LC) separation coupled with electrospray ionization (ESI) mass spectrometry has been used for the analysis of complex mixtures [23]. Many lipid species share similar *m/z* ratios and only the most abundant are generally detected [7,8,10–12,24] but the minor ones could also be relevant for physiological and pathological processes [25]. The use of very high mass resolution and accurate mass determination for MALDI MSI and LC-ESI-MS analyses allows resolving near isobaric lipid species for the detection of individual classes of phospholipids in a narrow mass range [19,26,27]. Moreover the high sensitivity and excellent (low) limit of detection of these types of techniques render possible the analysis of less-abundant lipids. These ones are indeed potential lipid markers of a disease and are quite difficult to be characterized in highly complex mixture [26].

**Figure 1.**
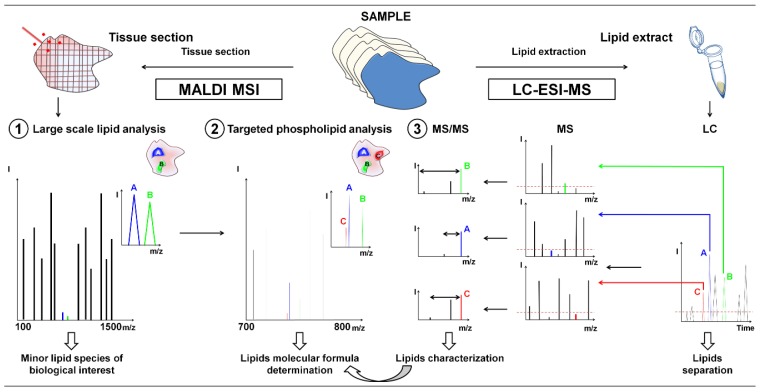
General workflow for the characterization by LC-ESI-MS guided by MALDI MSI of low-abundant lipids in tissue using FTICR mass spectrometry. (**1**) Large scale lipid analysis by MALDI MSI to target lipids of biological relevance after comparison with histochemical staining; (**2**) Targeted MALDI MSI analysis and molecular formula determination by exact mass measurement; and (**3**) LC-ESI-MS analyses for the characterization of low-abundant PL classes from lipid extracts.

In this study, high resolution mass spectrometry MALDI MSI coupled to histochemistry was used to localize and identify minor lipid species and further characterization was achieved by LC-ESI-MS (Figure 1). First, a MALDI MSI image of lipids was acquired in broadband detection mode and minor lipid species were selected to analyze tumor heterogeneity according to regions of interest associated with specific biological processes such as proliferating tumor area, necrosis, and inflammatory process on xenografts of human breast cancer cells, either highly invasive (MDA-MB-435 and MDA-MB-231 cells) or poorly aggressive (MCF-7 cells). The MALDI MSI analysis was then performed using the narrowband detection mode at higher mass resolution, in order to discriminate lipids with close masses. The high mass accuracy allowed the determination of their molecular formula by exact mass measurements. In the final step, LC-ESI-MS analysis was used to characterize the suspected phospholipids (PLs) from lipid extracts. We herein provide evidence for the suitability of this highly sensitive MSI based on molecular histology for the analysis of low-abundant PL species in different microenvironmental tumor regions of biological interest in human breast tumor xenografts.

## 2. Results and Discussion

### 2.1. *In Situ* Analysis by MALDI-FTICR MSI and Immunohistochemistry Reveal Specific Low-Abundant Lipid Species and Breast Tumor Heterogeneity

**Figure 2.**
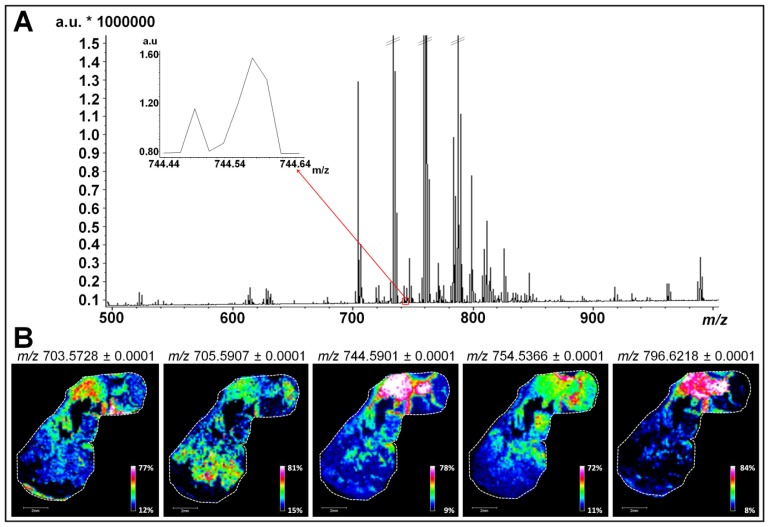
Large scale lipid analysis by MALDI MSI for low-abundant lipids of biological relevance targeting. (**A**) Broadband average mass spectrum of a simultaneous MALDI MSI analysis on sections of tumors derived from MCF-7, MDA-MB-231, and MDA-MB-435 cells. Inset indicates molecular species in a mass range comprised between *m/z* 744.44 and 744.64 considered for lipid mapping; and (**B**) MALDI MSI ion image representing the repartition of five low-abundant PLs (*m/z* 703.5728, *m/z* 705.5907, *m/z* 744.5901, *m/z* 754.5366 and *m/z* 796.6218) in a MDA-MB-435 tissue section (scale bars, 2 mm).

In order to investigate all lipid classes, tumor sections obtained from tumor xenografts of three different human breast cancer cell lines were firstly analyzed by MALDI MSI in a mass range comprised between *m/z* 100 and 1500. Results of Figures 2A and S1 correspond to the average mass spectrum obtained from the simultaneous analysis of sections of tumors derived from MCF-7, MDA-MB-231 and MDA-MB-435 cells. As shown in previous studies, the use of the 1,5-DAN matrix on tissue sections has proven efficacy for providing rich lipid signatures for MALDI MSI analysis in both negative and positive polarity with no analyte delocalization [28,29]. Data presented in Figures 2A and S1 show a typical MALDI-MSI lipid profile with peaks of high intensity characteristic of the different phospholipid (PL) species (between *m/z* 700 and 800) [30]. Most of these PLs belong to the phosphatidylcholine (PC) and phosphatidylethanolamine (PE) classes that are the most abundant PLs in mammalian cell membranes (40%–50% of total PLs for PC and 20%–50% for PE) [31]. The regulation of these PL species, notably in cellular membrane, has been associated with apoptosis [32], malignant transformation [33] and oxidative process [34]. In addition to the most abundant PC and PE detected, peaks with signal intensity barely detectable above the noise level are present and were considered for lipid mapping (Tables 1 and 2 and examples in insets of Figures 2A and S1).

**Figure 3.**
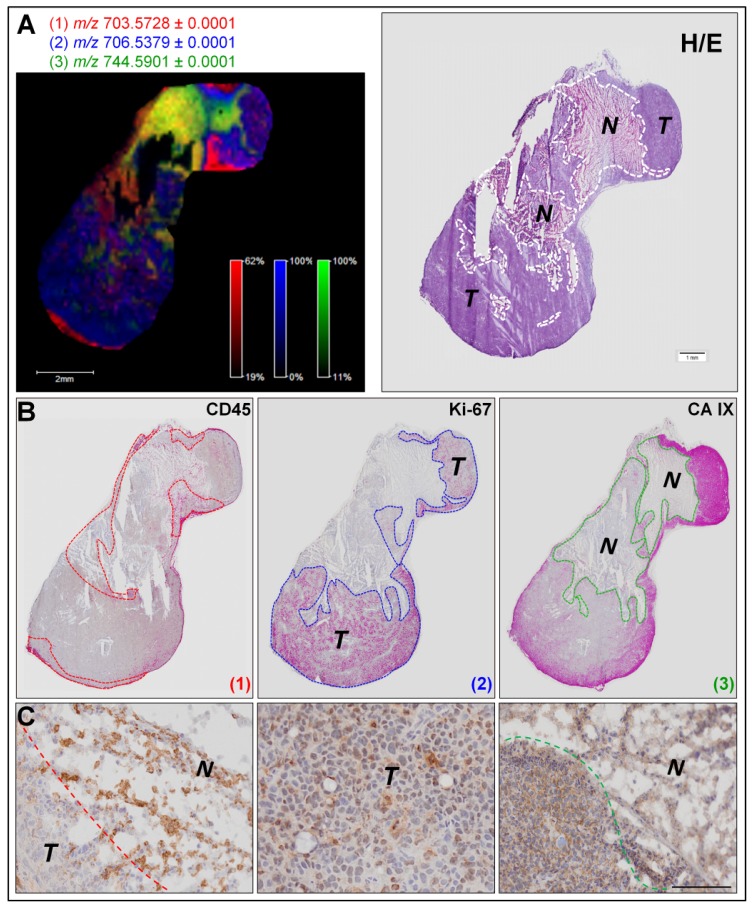
Determination of low-abundant PL species associated with different tumor compartments by correlation between MALDI MSI data and histochemical stainings. (**A**) MALDI MSI ion image representing the localization of three PLs (*m/z* values of 703.5728 in red, 706.5379 in blue, and 744.5901 in green) in a section of tumor induced by MDA-MB-435 cells (right panel) and hematoxylin/eosin staining (left panel). Dotted lines on the hematoxylin/eosin stained section image delineate necrosis “*N*” and tumor “*T*” areas; (**B**) Binary images of CD45 (left panel), Ki-67 (central panel) and CA IX (right panel) immunostainings of MDA-MB-435 serial tissue sections. Dotted lines of each binary image delineate the localization of PL (**1**) (*m/z* 703.5728 in red); PL (**2**) (*m/z* 706.5379 in blue) and PL (**3**) (*m/z* 744.5901 in green) shown in ion image. Squares indicate positions of high magnification microscopy images of immunohistochemistry (**C**); and (**C**) High magnification microscopy images of immunohistochemistry for CD45 (left), Ki-67 (middle) and CA IX (right) immunostainings (scale bars, 500 μm).

We then performed targeted MALDI MSI experiments of each tumor sections to focus on PL analysis. Ion images for *m/z* values of 703.5728, 705.5907, 744.5901, 754.5366, and 796.6218 revealed that numerous low-abundant PLs display a specific distribution within each section (as exemplified for MDA-MB-435 tumor section, Figure 2B). As shown in the left panels of Figures 3A and S2A, ion images (overlays of three ion images) fit with the general histological features revealed by H&E staining (right panels of Figures 3A and S2A for MDA-MB-435 and MCF-7 tumor sections, respectively). To determine if each minor PL is associated with different tumor compartments, we faced MALDI MSI ion images with binary images displaying three different immunostainings (CD45 positive inflammatory cells, Ki-67 positive proliferating human tumor cells and carbonic anhydrase IX positive corresponding to hypoxic areas) (Figure 3B,C for MDA-MB-435 and S2B for MCF-7 tumor section). In this way, we were able to confirm the biological relevance of the PLs that display a specific biological distribution in the areas of proliferating human tumor cells (*m/z* values of 706.5379), the hypoxia (*m/z* values of 744.5901, 754.5366, and 796.6218) and inflammation (*m/z* values of 703.5728 and 705.5907). Notably, such specific spatial distribution is not observed for other PLs present in the whole tissue section (as example PLs with an *m/z* value of 758.5703 and 760.5849, Figure S3). Additional illustrative examples of low-abundant PL species specifically associated with one or two/three tumor compartments are listed in Tables 1 and 2, respectively.

We herein demonstrate the suitability of PL identification to identify and/or delineate the different compartments composing the complex tumor ecosystem and which are known to influence the evolution of cancer and their response to therapeutic agents. The involvement of phospholipids in inflammatory processes, including their use by macrophages has already been reported [35]. Furthermore, oxidized PLs (oxPLs) are abundantly found at sites of inflammation and are considered to play an active role in the modulation of the immune response [36]. The relationship between active oxygen species, lipid peroxidation and necrosis has been highly demonstrated in many pathological processes [37–39].

Tumor hypoxia is an important factor influencing malignant progression and tumor cell sensitivity to radio- or chemotherapy treatment. Actually, a few markers of hypoxic zones such as carbonic anhydrase IX, glucose transporter-1 (GLUT-1) or HIF1α are available [40,41]. Consequently, a method that provides a highly sensitive MSI-based analysis of low-abundant PL species for concomitant detection and analysis of hypoxic areas and the surrounding tumor cells opens new perspectives for studying the tumor adaptation to hypoxic conditions and to predict their response to treatment.

### 2.2. Use of High-Resolution Mass Spectrometry for the Identification of Minor Lipid Species with Biological Relevance

**Figure 4.**
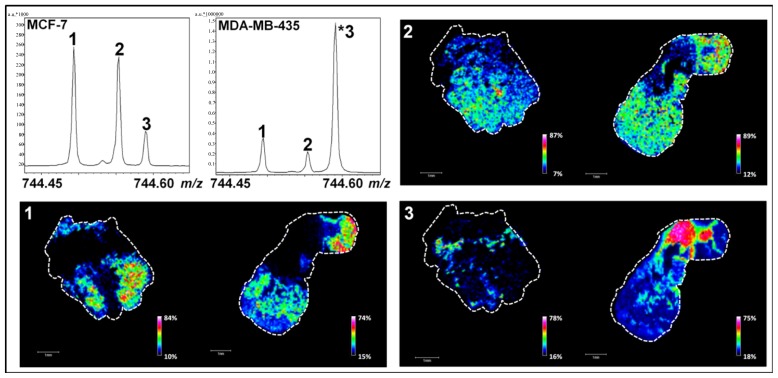
MALDI MSI analyses of MCF-7 (to the left) and MDA-MB-435 (to the right) tumor sections in narrowband mode. Associated ion images represent the localization of low-abundant PLs with *m/z* 744.49419 (peak 1), *m/z* 744.55385 (peak 2) and *m/z* 744.59019 (peak 3). PL relative contents determined by LC-ESI-MS are available in Table S3 (scale bars, 1 mm).

One of the main current issues in lipidomics is the accurate detection and localization of minor PL species with near isobaric masses. We herein demonstrate that one way of tackling this problem is the use of FTICR mass spectrometers with a suitable mass resolution to detect two or more (low-abundant) PLs within a very tight mass range. Our result indicates that in addition to the peak corresponding to a PL with an *m/z* value of 744.59019 (Figures 2B and 4, peak 3), two supplementary peaks were detected in a mass range that does not exceed 0.2 Th (e.g., *m/z* values of 744.49419, peak 1 and 744.55385, peak 2) (Figure 4). Other peaks with low *m/z* differences were also detected and another example is shown in Figure S4 (*m/z* values of 796.52524, peak 1; 796.58547, peak 2 and 796.62181, peak 3). Interestingly, the relative intensities of these peaks were reproducible between three biological replicates and define patterns that can be associated with the tumor type and/or the invasiveness (MCF7 *versus* MDA-MB-231 and MDA-MB-435 tumors) [arrows in Figure S5 for *m/z* 744.45 to 744.60 (A), *m/z* 796.50 and 796.65 (B), *m/z* 790.50 and 790.58 (C)]. Using probabilistic Latent Semantic Analysis (pLSA), we were able to confirm indirectly these specific patterns by visualizing the discriminating peaks between MCF7 *versus* MDA-MB-231 (Figure S6A) and MCF7 *versus* MDA-MB-435 tumors (Figure S6B). This result is in agreement with a recent study showing that the relative abundance of PCs varies according to the degree of differentiation and metastatic potential in breast cancer cells [42]. Studies have also evidenced that PL cell content can change in murine mammary tumor cells and human cancer [43,44] and is due to an enhanced cell membrane synthesis depending on cell growth phase, cell type and malignancy [45]. For PC and PE, variations are predominantly observed during the G1 phase and this is due to a higher activity of the enzymes controlling their metabolism [46]. The presence of different PL patterns was also reported in biological models of primary human fibroblasts and macrophages [47]. Ion images of Figures 4 and S4 are in good agreement with this last study since each PL is differently distributed on MDA-MB-435 and MCF-7 tissue sections.

The identification of these PL species was carried out by database matching using Lipid Maps database. The high mass accuracy measurement enabled the determination of the elemental composition of these biomolecules with accuracy better than 0.5 ppm (Tables 3 and 4). However, the high number of PL species sharing the same elemental composition did not allow their univocal identification and, as listed in Tables S1 and S2, several PCs or PEs were identified at the same molecular mass. This warrants caution on minor PL identification based on elemental formulas.

### 2.3. Targeted Characterization of Specific Low-Abundant Phospholipid Species in Complex Mixtures by LC-ESI-MS

Due to the dynamic behavior of cells, their exact lipid content is difficult to assess. Numerous metabolic pathways tightly regulate the lipid biosynthesis and turnover [48] and, even with a FTICR mass spectrometer, minor PL species cannot be directly identified by MALDI-MSI (Tables S1 and S2). As previously shown, the development of a method for their analysis remains a challenge [19]. LC-ESI-MS has proven to be a multidimensional approach with good sensitivity for the study of high-abundant lipids, especially PLs [49,50].

**Figure 5.**
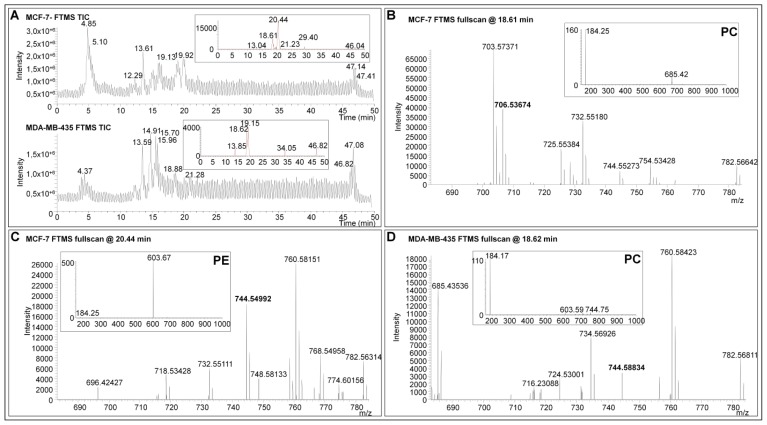
LC-ESI-MS analyses of lipid extracts from MCF-7 and MDA-MB-435 tissues. (**A**) Total ion currents (TIC) of LC-ESI-MS analyses. Insets show the extract ion currents ranging from *m/z* 744 to 745; and (**B**)–(**D**): Exact mass measurements acquired with the FTICR analyzer. Insets show MS/MS spectra at respective retention times. The visualization of an ion at *m/z* 184 indicates that the PL is a PC. Neutral loss fragment of 141 Da indicates that the PL is a PE.

To reduce the list of the potential candidates identified during MALDI MSI analyses, a hybrid linear ion trap/FTICR mass spectrometer was used for PL characterization by LC-ESI-MS. PL screenings were performed on complex total lipid extracts from MDA-MB-435 or MCF-7 xenografts using reverse phase high performance liquid chromatography. The separation of the isobaric species based on their different hydrophobic behavior allowed the characterization from complex samples of the lipid class of each minor PL analyzed by MALDI MSI (Figures 4 and S4). Assuming that the extraction methods and quantification of PL using the LC-ESI-MS method [51] is representative of the composition of the original PLs of the tissue (confirmed in Figure S7 for ions presented in Table 3), low-abundant PLs detected during MALDI FTICR MSI analyses were investigated by a targeted LC-ESI-MS screening (Figures 5A and S8A). The LC-ESI-MS extract ion currents (*m/z* ranges 744 to 745 and 796 to 797) are given in insets of Figures 5A and S8A. In this mass range, several chromatographic peaks associated to lipid species were revealed in each tissue. The exact mass measurements acquired with the FTICR analyzer allowed the assignment of the same PL species previously observed during MALDI MSI experiments (Figures 5B–D and S8B–D). All the isobaric PL species presented in Figures 4 and S4 were then characterized by MS/MS at respective retention times (insets of Figures 5B–D and S8–D). Collision induced dissociation of PC- and PE-derived [M + H]^+^ ions typically yield major product ions at *m/z* 184 or loss of 141 Th corresponding to the PE and PC polar headgroups, respectively [52]. Note that we roughly used a similar method for lipid identification as that from the recent paper from Kind *et al.*, except that we did not build any database for assisted identification using the NIST MS Search Program [53]. Elution time of lipids allowed the confirmation of the elemental composition of each PL of Tables 3 and 4 with regards to Na and K adducts. Moreover, ionic suppressive effects can be observed between PC and PE according to the matrix used during MALDI MSI analysis (data not shown). Due to the separation of PLs by LC-ESI-MS, interferences are not co-eluted with compounds of interest and avoid the suppression of ionization that usually occurs during the MALDI process [54,55]. The relative abundance of PL species can be assessed during LC-MS experiments. Nonetheless, the absolute quantification and unambiguous identification of PLs could be ensured using the appropriate internal standards. Thus, the use of LC-ESI-MS is mandatory to discriminate between high- and low PL species. The identification of PL species on the basis of the exact mass measurement during MALDI MSI did not reach the highest confidence level for the full characterization of PL species. Efficient ionization by LC-ESI-MS, using tandem MS (e.g., ion trap), high mass resolution and accurate mass analyzer (*i.e.*, FTICR or Orbitrap) allied to MS spectra obtained in parallel with MALDI MSI allowed the assignation of the sub-class of minor isobaric PLs on the basis of the identified head group by MS/MS (Tables S1 and S2). These data provide a proof-of-concept for the suitability of high resolution MS associated to immunohistochemical analysis to identify and localize low-abundant lipid species. In perspective, the use of appropriate (internal) standards will lead to the complete and unambiguous characterization of lipid isomers identified by LC-MS guided by high resolution MALDI imaging.

Breast cancer progression and metastatic dissemination is determined by the heterogeneity of both intrinsic breast cancer cell features and extreme factors related to the tumor microenvironments [56]. Among microenvironmental factors, oxygenation status (hypoxia and normoxia) and the recruitment of host cells including inflammatory, vascular cells or stroma cells are determinant. This complex tumoral ecosystem contributes to tumor sensitivity to radio- or chemotherapy and to the selection of aggressive tumor clones with metastatic potential. The heterogeneous nature of the tumor is highlighted in the present work by the detection of low-abundant PL species specifically associated with different tumor microenvironments. We observed a gradient of minor PL species from the center of the necrotic area to the proliferating tumor region (Figure S9) but none of the major PLs shared a similar distribution (as exemplified in Figure S3). This finding underlines the usefulness of developing high resolution MS methods to detect, separate and identify low-abundant PL species; potential markers of metabolic reprogrammation or regulators of malignant feature. A better molecular characterization of the different tumor compartments and of the tumor-host interface is mandatory to improve our understanding of complex biological processes occurring inside tumors, and to unravel molecular mechanisms associated with radio and/or chemo-resistance. In addition, cartography of low-abundant PL distribution among tumors will be helpful to investigate the metabolism of tumors, recently proposed by Hanahan *et al.* as a novel hallmark of cancer [57].

## 3. Experimental Section

### 3.1. Reagents

Purified matrix 1,5-diaminonaphtalene (1,5-DAN) was purchased from Sigma-Aldrich, Saint Louis, MO, USA. For LC-MS experiments, all chemicals were purchased from Sigma-Aldrich except ultra-pure water. Highly pure water of 18.2 MΩ-cm purity was produced using a Q-gard 1 purification pack (Millipore, Molsheim, France). Glass and polypropylene vials of 2 mL and 200 μL were purchased from VWR (VWR, Leuven, Belgium). For LC-ESI-MS experiments, the mobile phases were (A) 10 mM of ammonium acetate and 1 mM of acetic acid dissolved in 40% methanol and (B) 10 mM of ammonium acetate and 1 mM of acetic acid dissolved in 59.5% of isopropanol and 39.5% of methanol. Buffers (A) and (B) were freshly prepared by dilution of a stock solution of 1 M ammonium acetate and 0.1 M acetic acid solution in the appropriate volume of solvents.

### 3.2. Phospholipid Standards

Standards of PC(18:0/18:1) (1-stearoyl-2-oleoyl-*sn*-glycero-3-phosphocholine) (*M*_r_ 787.6091 Da), PC(16:0/14:0) (1-palmitoyl-2-myristoyl-*sn*-glycero-3-phosphocholine) (*M*_r_ 705.5308 Da), PE(18:0/18:1) (1-stearoyl-2-oleoyl-*sn*-glycero-3-phosphoethanolamine) (*M*_r_ 745.5621 Da) and PE(16:0/18:1) (1-palmitoyl-2-oleoyl-*sn*-glycero-3-phosphoethanolamine) (*M*_r_ 717.5308 Da) were purchased from Avanti Polar Lipids, Alabaster, AL, USA. All phospholipid standard solutions were prepared at a concentration of 25 mg/mL in MeOH/CHCl_3_ (50:50 *v*/*v*) and stored at a temperature of −20 °C.

### 3.3. Xenografts

Human breast cancer cells (MDA-MB-231, MDA-MB-435 and MCF-7) were cultured in Dulbecco’s Modified Eagle’s Medium (DMEM) supplemented with 10% fetal bovin serum (FBS), l-Glutamine (2 mM), penicillin (100 U/mL) and streptomycin (100 μg/mL) at 37 °C in a 5% CO_2_ humid atmosphere. All culture reagents were purchased from Gibco-Life Technologies (Invitrogen, Paisley, UK). For *in vivo* injection, subconfluent cells were trypsinized, resuspended in serum-free medium (5 × 10^6^ cells/mL) and mixed with an equal volume of cold Matrigel. Cell suspension (10^6^ cells/400 μL) was injected s.c. in both flanks of 6-month-old recombination Activating Gene RAG^−/−^ mice (provided by the Central Animal Housing of Liège University, Liège, Belgium). Note that all animal procedures were performed according to the Federation of European Laboratory Animal Sciences Associations (FELASA) and local ethical committee at University of Liege (Belgium). Animals were housed within the accredited animal facility of GIGA located at the CHU-Sart Tilman (University of Liege, Liege, Belgium). Five to six weeks post-injection, mice were sacrificed and the tumors were resected and snap frozen by immersion in pre-cooled isopentane at −80 °C for 1 min. Tumors were kept at −80 °C until use.

### 3.4. Tissue Preparation and Immunochemistry

Tissue sections were prepared using a Research Cryostat Leica CM3050 S (Leica Microsystems Wetzlar, Germany) with the microtome chamber chilled at −25 °C and the specimen holder at −15 °C, cryostat sections of 14 μm thick were mounted onto ITO-coated microscopic slides (Bruker Daltonics, Bremen, Germany) adapted for MALDI mass spectrometry and dried 2 h in a desiccator.

The following protocol for hematoxylin and eosin (H&E) staining was performed: (1) rehydrate in 100% and 95% EtOH (2 min each), (2) rinse in H_2_O (10 s), (3) stain in hematoxylin (CAT Hematoxylin) (3 min), (4) rince in tap H_2_O (10 s), (5) rince in 70% EtOH/1% HCl (20 s), (6) rince in H_2_O (15 min), (7) dehydrate in 95% EtOH (1 min), (8) counterstain in eosin (EDGAR DEGAS Eosin) (2 min), (9) rince and dehydrate in 95% EtOH (20 s), (10) rince and dehydrate again in 100% EtOH (20 s) and (11) dip in xylene (40 s). Sections were dried at room temperature in a hood and mounted with EUKITT (Kindler GmbH, Ziegelhofstrasse, Freiburg, Germany).

Immunostainings were performed with a mouse monoclonal anti-human-Ki-67 (DakoCytomation, Glostrup, Denmark) for proliferating tumor cells, a rat anti-CD45 coupled to biotin (BD Biosciences, Tennessee, CA, USA) for inflammatory cell and a rabbit anti-carbonic anhydrase IX (Abcam, Cambridge, UK) for hypoxia. Frozen sections were fixed in formol solution (4%) or in paraformaldehyde (PAF 4% at 4 °C) for anti-CD45 antibody. For antigen retrieval, sections were incubated 10 min in Triton/PBS solution (0.1%). After, endogenous peroxidase activity was blocked with H_2_O_2_ 3% (DakoCytomation), nonspecific binding was prevented by incubation of slides in PBS/BSA 10% (Fraction V, Acros Organics, NJ, USA) for 1 h for Ki 67 antibody and 30 min in normal swine serum (Fraction V, Acros Organics) for carbonic anhydrase IX antibody. Universal blocking reagent 10% (Biogenex HK085-5KE, Fremont, CA, USA) was used for sections stained with rat anti CD45/biotin.

Ki-67 labeling was performed by a 1 h incubation with antibody (1:500) raised against the human protein and revealed with the power vision poly-HRP anti-Mouse/Rabbit/Rat IgG kit (1/100, DakoCytomation). For carbonic anhydrase staining, slides were incubated overnight with antibodies (1:1000, diluted in PBS/Normal Goat Serum) at 4 °C and with the power vision poly-HRP anti-Mouse/Rabbit/Rat IgG kit (1/100, DakoCytomation). Slides were revealed with Vector DAB (SK-4100, Vector Laboratories, Burlingame, CA, USA).

For CD45 staining, slides were incubated with rat anti-CD45/biotin (BD Biosciences) (1:1500, diluted in PBS/BSA) overnight at 4 °C and incubated with the streptavidin poly-HRP kit (1/500, DakoCytomation, Glostrup, Denmark) for 30 min.

Virtual images were acquired with the fully automated digital microscopy system dotSlide (Olympus, BX51TF, Aartselaar, Belgium) coupled with a Peltier-cooled high resolution digital color camera (1376 × 1032 pixels) (Olympus, XC10, Aartselaar, Belgium). Digital images of the whole tissue sections were digitized at high magnification (60×) producing virtual images in which the pixel size is 10 μm. Image processing was performed using image analysis tool box of MATLAB 9.2 software (Matworks, Natick, MA, USA). In order to increase the contrast between the selected and surrounding regions, the excess of red component on images (two times red value minus blue value minus green value) was calculated.

### 3.5. MALDI Fourier Transform-Ion Cyclotron Resonance MSI Analyses

#### 3.5.1. Matrix Deposition

1,5-DAN deposition was performed in a homemade sublimation apparatus as previously described [28]. In the bottom of the sublimation apparatus 300 mg of 1,5-DAN matrix was deposited. Protocol was optimized for a fixed vacuum of 25 mTorr, monitoring temperature (150 °C), time of application (5 min), target plate temperature (14 °C) to ensure a homogeneous matrix coating.

#### 3.5.2. Mass Spectrometry

Mass spectra were acquired using a SolariX Fourier transform mass spectrometer (FTMS) (9.4 T) equipped with an ESI/MALDI Dual Ion Source including Smartbeam^TM^ II laser (BrukerDaltonics, Bremen, Germany). Images were acquired with a pixel step size for the surface raster set to 80 μm with FlexImaging 3.0 software (Bruker Daltonics, Bremen, Germany). Mass spectra were externally calibrated using a phospholipid standards solution (PC(18:0/18:1)/PC(16:0/14:0)/PE(18:0/18:1)/PE(16:0/18:1); 1:1:1:1 *v*/*v*/*v*/*v*). MALDI lipid mass spectra were acquired in positive ion mode from 100 laser shots that accumulated at each spot. The laser power was set to 18% with a frequency of 1000 Hz. For broadband detection mode analyses, mass range was set to *m/z* 100–1500 and time of flight value was 7 ms. Q1 mass was fixed at *m/z* 750 to focus on PL species, also taking into account other lipid species that can be detected during tissue MS analysis. Ion cooling time was set to 0.01 s. For narrowband detection mode analyses, center mass was set to *m/z* 745 ± 50 and time of flight value was 0.001 ms. For phospholipid molecular formula determination, monoisotopic masses from acquired mass spectra selected from images acquired from tumor sections were labeled using DataAnalysis 4.0 software (Bruker Daltonics, Bremen, Germany) with the FTMS peak-picking algorithm according to default parameters. Molecular formulas were determined manually using the SmartFormula algorithm in DataAnalysis 4.0 (Bruker Daltonics, Bremen, Germany). Tolerance was set to 1 ppm. Phospholipid identification was done by searches against “LIPID Metabolites and Pathways Strategy” (Lipid Maps) database (website: http://www.lipidmaps.org/) [58]. Identifications were performed by “Text-based searches” using experimental monoisotopic masses of each low-abundant PL species. Mass tolerance was set to ±0.01 Da. Molecular formulas were confirmed from ones determined with SmartFormula algorithm.

### 3.6. Probabilistic Latent Semantic Analysis

pLSA were carried out using ClinPro Tools 3.0 software (Bruker Daltonics, Bremen, Germany). Mass spectra from narrowband detection mode analyses of MCF-7, MDA-MB-231 and MDA-MB-435 samples were imported into ClinPro Tools 3.0 software. Normalization, peak peaking and spectra internal recalibration were automatically performed using the software. An average mass spectrum created from all single spectra was used for peak selection.

### 3.7. LC-ESI-MS Analyses

#### 3.7.1. Phospholipid Standard Preparations

Before injection in the High Performance Liquid Chromatography (HPLC) (Agilent, Waldbronn, Germany) column, each phospholipid standard (10 μL) was mixed in 160 μL of a solution composed of 40% mobile phase (B) and 60% mobile phase (A).

#### 3.7.2. Lipid Extraction from MDA-MB-231, MDA-MB-435, and MCF-7 Tumors

Lipids were extracted according to the Folch *et al.* method [59]. Amounts of 100 mg of each tumor were homogenized in MeOH/CHCl_3_ (2:1 *v*/*v*) to a final dilution 20-fold the volume of the tissue sample. After homogenization, mixtures were agitated during 50 s at 3000 *g* in a MagNaLyser Instrument (Roche, Basel, Switzerland) at room temperature (RT) and stored at 4 °C for 5 min. This step was repeated twice. Samples were then centrifuged (50 s at 3500 *g* at RT) to recover the liquid phase. The supernatant was washed with 0.2 volumes of H_2_O milliQ and briefly vortexed. The final biphasic mixtures were centrifuged (10 min, 2000 *g*) and the upper aqueous phase discarded. The lower chloroform phase of each sample was evaporated under vacuum in a SPD121Savant SpeedVac rotary evaporator (Thermo Electron Corporation, Waltham, MA, USA). Following the Folch extraction, 50 mg of each dry sample was recovered. Samples were then dissolved in 500 μL of a mixture of MeOH/CHCl_3_ (50:50 *v*/*v*). Each lipid extract (20 μL) was dissolved in 180 μL of a mixture of 40% of mobile phase (B), 60% of mobile phase (A) for LC-ESI-MS analyses.

#### 3.7.3. LC-ESI-MS Analyses

Phospholipid separation and identification were performed using the method published by Uhl *et al.* [51] with modifications. The HPLC system was an Agilent 1100 (Agilent Technologies, Takakuramachi, Japan) series equipped with a quaternary HPLC pump and an on-line membrane degasser. For the autosampler, we employed the standard Agilent autosampler Automatic Liquid Sampler (ALS) using the 100 vials system and a 100 μL variable volume sampling loop with a thermostat system set at 6 °C. The column oven was also available and the temperature oven was fixed at 60 °C. The RP-HPLC column was a C18 Xbridge column of 4.6 mm × 150 mm × 3.5 μm from Waters (Waters, Milford, MA, England). The mass spectrometer was a LTQ FTICR Ultra (Thermo Finnigan, Waltham, MA, USA), a hybrid mass spectrometer using a linear quadrupole ion trap and an Ion Cyclotron Resonance (ICR) mass analyzer (7 T magnet). The ion source was the Ion Max electrospray source operating in positive mode. The instrument was first tuned using the recommendation of the manufacturer. Then ion optic was optimized by direct infusion of the phospholipid standards. The Fourier Transform mass spectrometer (FTMS) was mass calibrated using some phosphoric acid solution at 0.1% in solution in 50% acetonitrile (Sigma-Aldrich, St. Louis, MO, USA).

A sample (20 μL) was injected for each LC-ESI-MS acquisition. The needle was rinsed using a mix of 90% (B) and 10% (A) after injection. The flow rate of the mobile phase was 0.8 mL/min. The elution program was first 40% of (B) over 4 min. Then the proportion of (B) was linearly increased until 70% over 6 min, then the proportion of (B) was slowly increased to achieve 99% using a linear gradient over 25 min and maintained for 6 min. Finally the proportion of (B) was linearly decreased to 40% in 1 min and maintained for 8 min before a new injection. A new sample could be injected after 50 min. A blank sample was periodically injected between each sample. The outlet of the HPLC column was connected to the source of the mass spectrometer. A micro-splitter (Supelco, Sigma-Aldrich) was used in order to obtain a flow rate of 60 μL/min entering the ESI source of the mass spectrometer. The ESI source voltage was +2.0 kV, the sheath gas flow was fixed to 50 (arbitrary unit). The temperature of the transfer capillary was set to 220 °C where a voltage of 17 V was applied. The tube lens voltage was fixed to 70 V. The ion optic was set using the following values: Multipole off set 00: −4.25 V; Lens 0 voltage: −4.0 V; Multipole: −4.75 V, Lens 1: −9.0 V; Gate lens: −78 V; multipole 1: −7.0 V; Multipole RF: 400 Vpp and Front lens: −5.25 V. A 50 min segment composed of nine event scans was used. The first event scan was a fullscan spectrum over a mass range of *m/z* 100–1000 using the linear quadrupole ion trap MS. The maximum filling time was fixed to 200 ms and five microscans were used. The second scan event was similar to the first scan event except that FTMS was used. The resolution was set to 25,000 and the maximum filling time was fixed to 200 ms. The FTMS scan event maximum duration was about 2.5 s. The four next scan events were MS/MS experiments using Collision Induced Dissociation (CID) ion activation at *m/z* ratios 744.5, 766.5, 790.5 and 796.5 using a selection window mass range of 2 Th and normalized collision energy of 25%. The mass ranges for these spectra were about *m/z* 170–1000. For this purpose, the Act Q was fixed to 0.20 and the act time was fixed to 30 ms. Xcalibur v2.0.7 (Thermo Fisher Scientific) was used for data acquisition and processing. The mass spectra were recalibrated off line on the basis of the PC(18:0/18:2) mass observed in each lipid extract. PC(18:0/18:2) is an ubiquitous PL and notably detected in all analyzed tissues by LC/MS (*i.e.*, MCF7, MDA-MB-231 and MDA-MB-435 xenografts, data not shown) and MALDI MSI (as example in Figures S10A,B for MCF7 and MDA-MB-435 tumor sections, respectively). Relative PL abundance was estimated by comparing the intensity of an ion of interest (*i.e*., *m/z* 744.49419, 744.55385 and 744.59019 from LC-ESI-MS analyses of MCF-7 and MDA-MB-435 tumor sections, Table S3) with the intensity of the most abundant ion observed in the base peak chromatogram. The PLs of interest were found to have less than 1% abundance compared to the most abundant species (*i.e*., eXtract Ion Chromatograms (XIC) ranged from *m/z* values 744.5 to 744.6 from LC-ESI-MS analyses of MCF-7, MDA-MB-231 and MDA-MB-435 tumor sections, Figure S7).

## 4. Conclusions

Histochemical analyses of human xenograft tissue sections combined with high mass resolution MALDI MSI brings a real advantage in lipidomics by allowing the characterization of low-abundant PL species and their localization in regions of biological relevance such as necrosis, inflammation and proliferating tumor cells. Moreover, the targeted analysis of the lipid total extracts by LC-ESI-MS guided by MALDI MSI allows a complementary identification of the minor PL markers. So far, the involvement of these low PLs in biological signaling processes has been overlooked due to technical limitations. Our study provides the first visualization and characterization of minor phospholipids in human breast tumor xenograft models. This innovative combination of methods paves the way for future comprehensive analyses of low-abundant lipids in various types of tissues and will enlighten processes occurring during tumor growth and death, survival and the inflammatory process. Local extraction of lipids for further HPLC-MS measurements on regions of interest will be the next step thereby allowing quantification.

## Supplementary Information



## Figures and Tables

**Table 1.** Low-abundant PL species associated with one specific tumor area detected during MALDI MSI analyses. Grey highlighted (+) corresponds to the localization of the PL species in tissues.

*m/z*_obs_	Necrosis	Inflammation	Tumor
703.5728	−	+	−
705.5821	+	−	−
706.5379	−	−	+
718.5748	+	−	−
720.5812	+	−	−
725.5574	+	−	−
728.5197	−	+	−
739.4666	−	−	+
740.4714	−	−	+
740.4993	−	−	+
740.5207	+	−	−
740.5573	+	−	−
741.5311	−	−	+
744.5512	−	−	+
744.5901	+	−	−
765.4836	−	−	+
768.5901	+	−	−
770.5107	−	−	+
772.5248	−	−	+
772.6217	+	−	−
774.6378	+	−	−
790.5098	−	−	+
790.5742	+	−	−
796.5252	−	−	+
796.5854	−	+	−
796.6218	+	−	−
798.5416	−	−	+

**Table 2.** Low-abundant PL species associated with two or three specific tumor areas detected during MALDI MSI analyses. Grey highlighted (+) corresponds to the localization of the PL species in tissues.

*m/z*_obs_	Necrosis	Inflammation	Tumor
704.5221	−	+	+
705.5904	+	−	+
720.5542	−	+	+
723.4943	+	−	+
730.5384	−	+	+
731.6066	+	+	+
734.5693	−	+	+
746.5698	+	+	+
746.6065	+	−	+
754.5364	+	+	+
772.5847	+	+	+
784.5725	+	+	−
784.5853	−	+	+
790.5419	+	+	−
794.6049	+	+	−

**Table 3.** Elemental formulas of minor PL species (*m/z* 744.49419, 744.55385, and 744.59019) detected during MALDI MSI analyses.

*m/z*_obs_	Elemental formula 1	*m*/*z*_th_	Error (ppm)
744.49419	C_38_H_76_NO_8_P ([M + K]^+^)	744.49401	0.24
744.55385	C_41_H_79_NO_8_P ([M + H]^+^)	744.55378	0.09
744.59019	C_42_H_83_NO_7_P ([M + H]^+^)	744.59017	0.04

1 Elemental formula of protonated ([M + H]^+^) and adducted ([M + K]^+^) ion species.

**Table 4.** Elemental formulas of minor PL species (*m/z* 796.52524, 796.58547, and 796.62181) detected during MALDI MSI analyses.

*m/z*_obs_	Molecular formula 1	*m*/*z*_th_	Error (ppm)
796.52524	C_42_H_80_NO_8_P ([M + K]^+^)	796.52531	0.1
796.58547	C_45_H_83_NO_8_P ([M + H]^+^)	796.58508	0.5
796.62181	C_46_H_87_NO_7_P ([M + H]^+^)	796.62147	0.4

1 Elemental formulas of protonated ([M + H]^+^) and adducted ([M + K]^+^) ion species.
