# Supplementary material for: Towards Lipidomics of Low-Abundant Species for Exploring Tumor Heterogeneity Guided by High-Resolution Mass Spectrometry Imaging
Source: Int J Mol Sci. 2013 Dec 17;14(12):24560–80. doi: 10.3390/ijms141224560 (PMC3876128; doi:10.3390/ijms141224560)
Supplement: Supplementary file 1 [file ijms-14-24560-s002.pdf]

Supplementary Information

Figure S1. MALDI MSI analyses of MCF-7, MDA-MB-231 and MDA-MB-435 tumor sections in broadband mode. Inset indicates minor molecular species in a mass range comprised between *m/z* values 796.46 and 796.64 considered for lipid mapping.

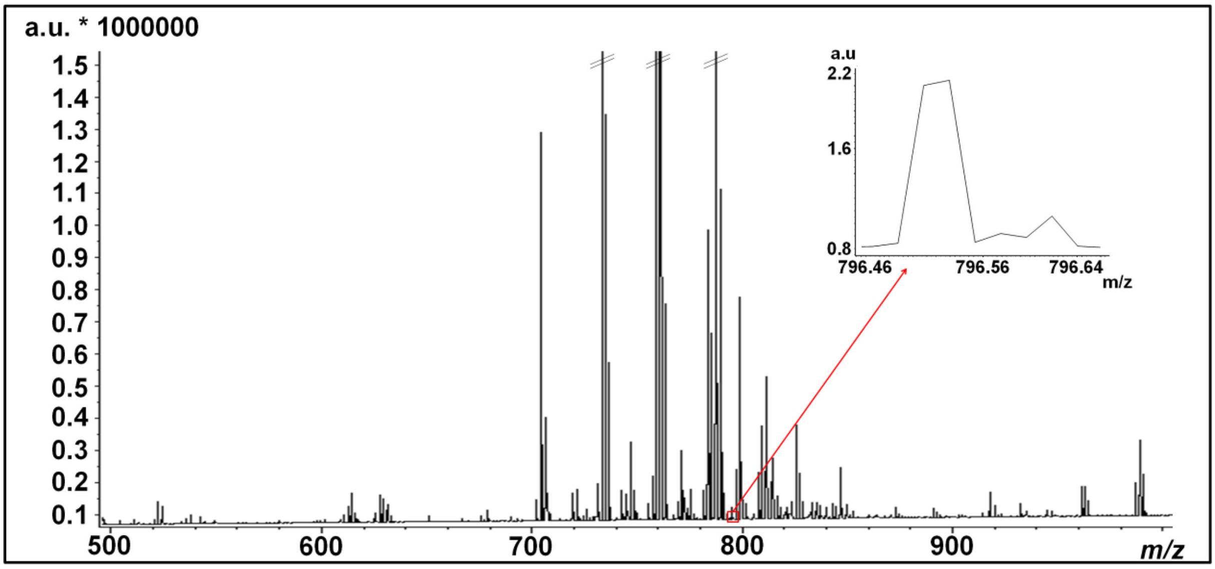

Figure S2. Determination of low-abundant PL species associated with different tumor compartments by correlation between MALDI MSI data and histochemical stainings. (**A**) MALDI MSI ion image representing the localization of three PLs (*m/z* values of 703.5728 in red, 706.5379 in blue and 796.6218 in green) in a section of tumor induced by MCF-7 cells (right panel) and hematoxylin/eosin staining (left panel). Dotted lines on hematoxylin/eosin stained section delineate necrosis "*N*" and tumor "*T*" areas. And (**B**) Binary images of CD45 (right panel), Ki-67 (central panel) and CA IX (left panel) immunostainings of MCF-7 serial tissue sections. Dotted lines of each binary image delineate the localization of PL (1) (*m/z* value of 703.5728 in red), PL (2) (*m/z* value of 706.5379 in blue) and PL (3) (*m/z* value of 796.6218 in green) shown in ion image.

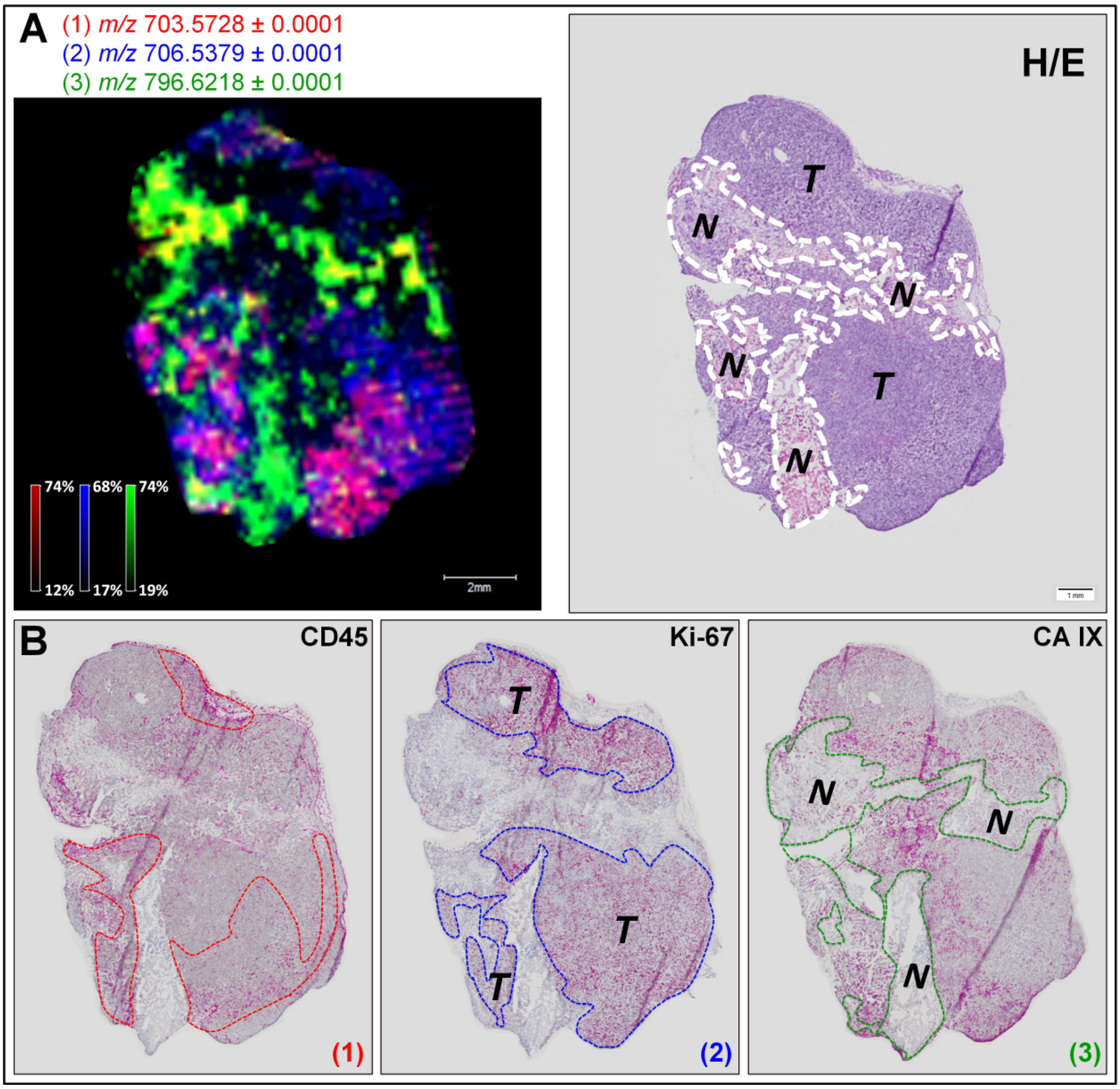

Figure S3. Localization of major PL species in MDA-MB-435 tumor sections. MALDI MSI ion images representing the localization of four major PL species (*m/z* values of 732.5513, 758.5703, 760.5849 and 786.6012) in a section of tumor induced by MDA-MB-435 cells.

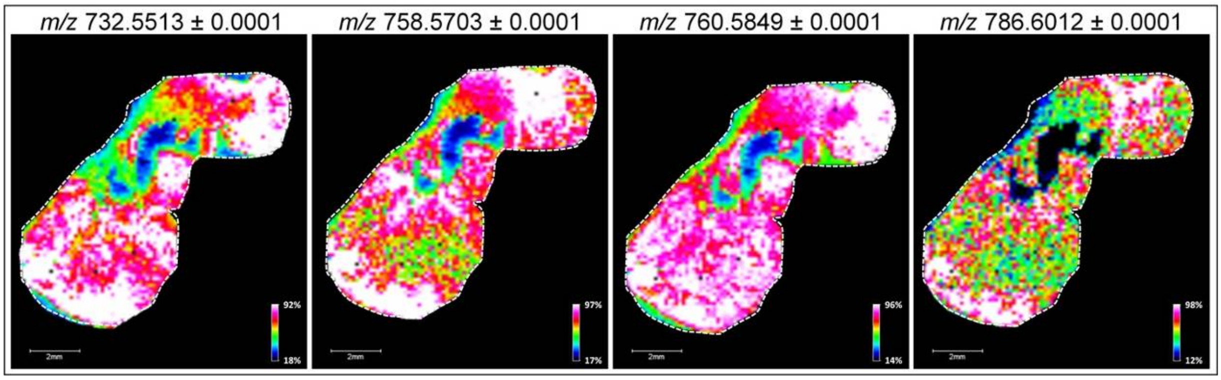

Figure S4. MALDI MSI analyses of MCF-7 and MDA-MB-435 tumor sections in narrowband mode. Associated ion images represent the localization of low-abundant PLs with *m/z* values of 796.52524 (peak 1), 796.58547 (peak 2) and 796.62181 (peak 3).

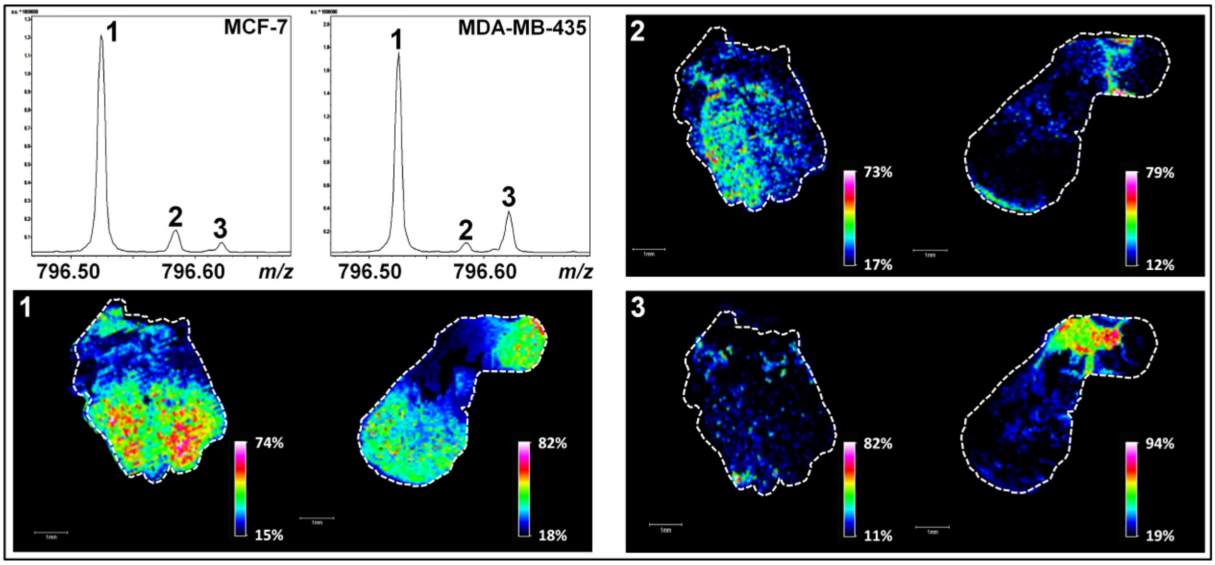

Table S1. Low-abundant PL species identified from elemental formula of Table 3 by database searching in lipidmaps.

M (Da)	LM-ID [1]	Common names	Formulas	Classes [2]
705.53030	LMGP01010395	PC(10:0/20:0)	$C_{38}H_{76}NO_8P$	PC
	LMGP01010416	PC(11:0/19:0)	$C_{38}H_{76}NO_8P$	PC
	LMGP01010438	PC(12:0/18:0)	$C_{38}H_{76}NO_8P$	PC
	LMGP01010461	PC(13:0/17:0)	$C_{38}H_{76}NO_8P$	PC
	LMGP01010481	PC(14:0/16:0)	$C_{38}H_{76}NO_8P$	PC
	LMGP01010530	PC(15:0/15:0)	$C_{38}H_{76}NO_8P$	PC
	LMGP01010560	PC(16:0/14:0)	$C_{38}H_{76}NO_8P$	PC
	LMGP01010702	PC(17:0/13:0)	$C_{38}H_{76}NO_8P$	PC
	LMGP01010736	PC(18:0/12:0)	$C_{38}H_{76}NO_8P$	PC
	LMGP01010969	PC(19:0/11:0)	$C_{38}H_{76}NO_8P$	PC
	LMGP01010995	PC(20:0/10:0)	$C_{38}H_{76}NO_8P$	PC
	LMGP01011265	PC(9:0/21:0)	$C_{38}H_{76}NO_8P$	PC
	LMGP02010337	PE-NMe(16:0/16:0)	$C_{38}H_{76}NO_8P$	PE
	LMGP02010377	PE(12:0/21:0)	$C_{38}H_{76}NO_8P$	PE
	LMGP02010413	PE(14:0/19:0)	$C_{38}H_{76}NO_8P$	PE
	LMGP02010542	PE(17:0/16:0)	$C_{38}H_{76}NO_8P$	PE
	LMGP02010623	PE(18:0/15:0)	$C_{38}H_{76}NO_8P$	PE
	LMGP02011170	PE(21:0/12:0)	$C_{38}H_{76}NO_8P$	PE
	LMGP02011181	PE(20:0/13:0)	$C_{38}H_{76}NO_8P$	PE
	LMGP02011186	PE(19:0/14:0)	$C_{38}H_{76}NO_8P$	PE
	LMGP02011227	PE(16:0/17:0)	$C_{38}H_{76}NO_8P$	PE
	LMGP02011233	PE(15:0/18:0)	$C_{38}H_{76}NO_8P$	PE
	LMGP02011253	PE(13:0/20:0)	$C_{38}H_{76}NO_8P$	PE
743.54595	LMGP01010543	PC(15:0/18:2(9Z,12Z))	$C_{41}H_{78}NO_8P$	PC
	LMGP01011354	PC(13:0/20:2(11Z,14Z))	$C_{41}H_{78}NO_8P$	PC
	LMGP01011396	PC(14:1(9Z)/19:1(9Z))	$C_{41}H_{78}NO_8P$	PC
	LMGP01011443	PC(15:1(9Z)/18:1(9Z))	$C_{41}H_{78}NO_8P$	PC
	LMGP01011465	PC(16:0/17:2(9Z,12Z))	$C_{41}H_{78}NO_8P$	PC
	LMGP01011481	PC(16:1(9Z)/17:1(9Z))	$C_{41}H_{78}NO_8P$	PC
	LMGP01011528	PC(17:1(9Z)/16:1(9Z))	$C_{41}H_{78}NO_8P$	PC
	LMGP01011557	PC(17:2(9Z,12Z)/16:0)	$C_{41}H_{78}NO_8P$	PC
	LMGP01011599	PC(18:1(9Z)/15:1(9Z))	$C_{41}H_{78}NO_8P$	PC
	LMGP01011618	PC(18:2(9Z,12Z)/15:0)	$C_{41}H_{78}NO_8P$	PC
	LMGP01011759	PC(19:1(9Z)/14:1(9Z))	$C_{41}H_{78}NO_8P$	PC
	LMGP01011836	PC(20:2(11Z,14Z)/13:0)	$C_{41}H_{78}NO_8P$	PC
	LMGP02010039	PE(18:1(9E)/18:1(9E))	$C_{41}H_{78}NO_8P$	PE
	LMGP02010044	PE(18:0/18:2(9Z,12Z))	$C_{41}H_{78}NO_8P$	PE
	LMGP02010052	PE(18:1(9Z)/18:1(9Z))	$C_{41}H_{78}NO_8P$	PE
	LMGP02010109	PE(18:1(6Z)/18:1(6Z))	$C_{41}H_{78}NO_8P$	PE
	LMGP02010420	PE(14:0/22:2(13Z,16Z))	$C_{41}H_{78}NO_8P$	PE
	LMGP02010448	PE(14:1(9Z)/22:1(11Z))	$C_{41}H_{78}NO_8P$	PE

Table S1. *Cont.*

M (Da)	LM-ID [1]	Common names	Formulas	Classes [2]
743.54595	LMGP02010510	PE(16:0/20:2(11Z,14Z))	$C_{41}H_{78}NO_8P$	PE
	LMGP02010530	PE(16:1(9Z)/20:1(11Z))	$C_{41}H_{78}NO_8P$	PE
	LMGP02010578	PE(17:1(9Z)/19:1(9Z))	$C_{41}H_{78}NO_8P$	PE
	LMGP02010607	PE(17:2(9Z,12Z)/19:0)	$C_{41}H_{78}NO_8P$	PE
	LMGP02010774	PE(19:0/17:2(9Z,12Z))	$C_{41}H_{78}NO_8P$	PE
	LMGP02010802	PE(19:1(9Z)/17:1(9Z))	$C_{41}H_{78}NO_8P$	PE
	LMGP02010848	PE(20:1(11Z)/16:1(9Z))	$C_{41}H_{78}NO_8P$	PE
	LMGP02010877	PE(20:2(11Z,14Z)/16:0)	$C_{41}H_{78}NO_8P$	PE
	LMGP02011043	PE(22:1(11Z)/14:1(9Z))	$C_{41}H_{78}NO_8P$	PE
	LMGP02011073	PE(22:2(13Z,16Z)/14:0)	$C_{41}H_{78}NO_8P$	PE
	LMGP02011193	PE(18:2(9Z,12Z)/18:0)	$C_{41}H_{78}NO_8P$	PE
743.58234	LMGP01020039	PC(O-16:0/18:2(9Z,12Z))	$C_{42}H_{82}NO_7P$	PC
	LMGP01030006	PC(P-16:0/18:1(9Z))	$C_{42}H_{82}NO_7P$	PC
	LMGP01030053	PC(P-18:0/16:1(9Z))	$C_{42}H_{82}NO_7P$	PC
	LMGP01030077	PC(P-20:0/14:1(9Z))	$C_{42}H_{82}NO_7P$	PC
	LMGP01030128	PC(P-16:0/18:1(11Z))	$C_{42}H_{82}NO_7P$	PC
	LMGP01030134	PC(P-18:1(11Z)/16:0)	$C_{42}H_{82}NO_7P$	PC
	LMGP01030144	PC(P-18:1(9Z)/16:0)	$C_{42}H_{82}NO_7P$	PC
	LMGP01090006	PC(16:0/P-18:1(11Z))	$C_{42}H_{82}NO_7P$	PC
	LMGP01090007	PC(16:0/P-18:1(9Z))	$C_{42}H_{82}NO_7P$	PC
	LMGP01090009	PC(16:1(9Z)/P-18:0)	$C_{42}H_{82}NO_7P$	PC
	LMGP01090012	PC(18:1(11Z)/P-16:0)	$C_{42}H_{82}NO_7P$	PC
	LMGP02020070	PE(O-20:0/17:2(9Z,12Z))	$C_{42}H_{82}NO_7P$	PE
	LMGP02030051	PE(P-18:0/19:1(9Z))	$C_{42}H_{82}NO_7P$	PE
	LMGP02030071	PE(P-20:0/17:1(9Z))	$C_{42}H_{82}NO_7P$	PE

Note: [1] Lipidmaps accession numbers. [2] PC (phosphatidylcholine) and PE (phosphatidylethanolamine) PL classes. Grey highlighted lines corresponds to the PL species characterized by LC-ESI-MS/MS analyses.

Table S2. Low-abundant PL species identified from elemental formula of Table 4 by database searching in lipidmaps.

M (Da)	LM-ID [1]	Common names	Formulas	Classes [2]
757.56160	LMGP01010585	PC(16:0/18:2(10E,12Z))	$C_{42}H_{80}NO_8P$	PC
	LMGP01010586	PC(16:0/18:2(11Z,13Z))	$C_{42}H_{80}NO_8P$	PC
	LMGP01010587	PC(16:0/18:2(2E,4E))	$C_{42}H_{80}NO_8P$	PC
	LMGP01010588	PC(16:0/18:2(2Z,4Z))	$C_{42}H_{80}NO_8P$	PC
	LMGP01010589	PC(16:0/18:2(6Z,9Z))	$C_{42}H_{80}NO_8P$	PC
	LMGP01010590	PC(16:0/18:2(9E,11E))	$C_{42}H_{80}NO_8P$	PC
	LMGP01010591	PC(16:0/18:2(9E,11Z))	$C_{42}H_{80}NO_8P$	PC
	LMGP01010592	PC(16:0/18:2(9E,12E))	$C_{42}H_{80}NO_8P$	PC
	LMGP01010594	PC(16:0/18:2(9Z,12Z))	$C_{42}H_{80}NO_8P$	PC
	LMGP01010678	PC(16:1(2Z)/18:1(9Z))	$C_{42}H_{80}NO_8P$	PC
	LMGP01010687	PC(16:1(9Z)/18:1(11Z))	$C_{42}H_{80}NO_8P$	PC

Table S2. *Cont.*

M (Da)	LM-ID [1]	Common names	Formulas	Classes [2]
757.56160	LMGP01010688	PC(16:1(9Z)/18:1(9Z))	$C_{42}H_{80}NO_8P$	PC
	LMGP01010727	PC(17:1(10Z)/17:1(10Z))	$C_{42}H_{80}NO_8P$	PC
	LMGP01010728	PC(17:1(9Z)/17:1(9Z))	$C_{42}H_{80}NO_8P$	PC
	LMGP01010745	PC(18:0/16:2(2E,4E))	$C_{42}H_{80}NO_8P$	PC
	LMGP01010585	PC(16:0/18:2(10E,12Z))	$C_{42}H_{80}NO_8P$	PC
	LMGP01010586	PC(16:0/18:2(11Z,13Z))	$C_{42}H_{80}NO_8P$	PC
795.57726	LMGP01010003	PC(17:0/20:4(5Z,8Z,11Z,14Z))	$C_{45}H_{82}NO_8P$	PC
	LMGP01011429	PC(15:0/22:4(7Z,10Z,13Z,16Z))	$C_{45}H_{82}NO_8P$	PC
	LMGP01011542	PC(17:1(9Z)/20:3(8Z,11Z,14Z))	$C_{45}H_{82}NO_8P$	PC
	LMGP01011571	PC(17:2(9Z,12Z)/20:2(11Z,14Z))	$C_{45}H_{82}NO_8P$	PC
	LMGP01011658	PC(18:3(6Z,9Z,12Z)/19:1(9Z))	$C_{45}H_{82}NO_8P$	PC
	LMGP01011687	PC(18:3(9Z,12Z,15Z)/19:1(9Z))	$C_{45}H_{82}NO_8P$	PC
	LMGP01011717	PC(18:4(6Z,9Z,12Z,15Z)/19:0)	$C_{45}H_{82}NO_8P$	PC
	LMGP01011742	PC(19:0/18:4(6Z,9Z,12Z,15Z))	$C_{45}H_{82}NO_8P$	PC
	LMGP01011770	PC(19:1(9Z)/18:3(6Z,9Z,12Z))	$C_{45}H_{82}NO_8P$	PC
	LMGP01011771	PC(19:1(9Z)/18:3(9Z,12Z,15Z))	$C_{45}H_{82}NO_8P$	PC
	LMGP01011845	PC(20:2(11Z,14Z)/17:2(9Z,12Z))	$C_{45}H_{82}NO_8P$	PC
	LMGP01011875	PC(20:3(8Z,11Z,14Z)/17:1(9Z))	$C_{45}H_{82}NO_8P$	PC
	LMGP01011904	PC(20:4(5Z,8Z,11Z,14Z)/17:0)	$C_{45}H_{82}NO_8P$	PC
	LMGP01012070	PC(22:4(7Z,10Z,13Z,16Z)/15:0)	$C_{45}H_{82}NO_8P$	PC
	LMGP02010677	PE(18:2(9Z,12Z)/22:2(13Z,16Z))	$C_{45}H_{82}NO_8P$	PE
	LMGP02010706	PE(18:3(6Z,9Z,12Z)/22:1(11Z))	$C_{45}H_{82}NO_8P$	PE
	LMGP02010734	PE(18:3(9Z,12Z,15Z)/22:1(11Z))	$C_{45}H_{82}NO_8P$	PE
	LMGP02010764	PE(18:4(6Z,9Z,12Z,15Z)/22:0)	$C_{45}H_{82}NO_8P$	PE
	LMGP02010862	PE(20:1(11Z)/20:3(8Z,11Z,14Z))	$C_{45}H_{82}NO_8P$	PE
	LMGP02010892	PE(20:2(11Z,14Z)/20:2(11Z,14Z))	$C_{45}H_{82}NO_8P$	PE
	LMGP02010922	PE(20:3(8Z,11Z,14Z)/20:1(11Z))	$C_{45}H_{82}NO_8P$	PE
	LMGP02010951	PE(20:4(5Z,8Z,11Z,14Z)/20:0)	$C_{45}H_{82}NO_8P$	PE
	LMGP02011027	PE(22:0/18:4(6Z,9Z,12Z,15Z))	$C_{45}H_{82}NO_8P$	PE
	LMGP02011054	PE(22:1(11Z)/18:3(6Z,9Z,12Z))	$C_{45}H_{82}NO_8P$	PE
	LMGP02011055	PE(22:1(11Z)/18:3(9Z,12Z,15Z))	$C_{45}H_{82}NO_8P$	PE
	LMGP02011084	PE(22:2(13Z,16Z)/18:2(9Z,12Z))	$C_{45}H_{82}NO_8P$	PE
	LMGP02011113	PE(22:4(7Z,10Z,13Z,16Z)/18:0)	$C_{45}H_{82}NO_8P$	PE
	LMGP02011177	PE(20:0/20:4(5Z,8Z,11Z,14Z))	$C_{45}H_{82}NO_8P$	PE
	LMGP02011200	PE(18:0/22:4(7Z,10Z,13Z,16Z))	$C_{45}H_{82}NO_8P$	PE
795.61364	LMGP01020100	PC(O-18:0/20:4(5E,8E,11E,14E))	$C_{46}H_{86}NO_7P$	PC
	LMGP01020102	PC(O-18:0/20:4(5Z,8Z,11Z,14Z))	$C_{46}H_{86}NO_7P$	PC
	LMGP01020192	PC(O-16:0/22:4(7Z,10Z,13Z,16Z))	$C_{46}H_{86}NO_7P$	PC
	LMGP01020231	PC(O-20:0/18:4(6Z,9Z,12Z,15Z))	$C_{46}H_{86}NO_7P$	PC
	LMGP01020247	PC(O-18:0/20:4(8Z,11Z,14Z,17Z))	$C_{46}H_{86}NO_7P$	PC
	LMGP01030067	PC(P-18:0/20:3(8Z,11Z,14Z))	$C_{46}H_{86}NO_7P$	PC

Table S2. *Cont.*

M (Da)	LM-ID [1]	Common names	Formulas	Classes [2]
795.61364	LMGP01030088	PC(P-20:0/18:3(6Z,9Z,12Z))	$C_{46}H_{86}NO_7P$	PC
	LMGP01030089	PC(P-20:0/18:3(9Z,12Z,15Z))	$C_{46}H_{86}NO_7P$	PC
	LMGP01030131	PC(P-18:0/20:3(5Z,8Z,11Z))	$C_{46}H_{86}NO_7P$	PC
	LMGP01030138	PC(P-18:1(11Z)/20:2(11Z,14Z))	$C_{46}H_{86}NO_7P$	PC
	LMGP01090038	PC(20:2(11Z,14Z)/P-18:1(11Z))	$C_{46}H_{86}NO_7P$	PC
	LMGP01090039	PC(20:2(11Z,14Z)/P-18:1(9Z))	$C_{46}H_{86}NO_7P$	PC
	LMGP01090041	PC(20:3(5Z,8Z,11Z)/P-18:0)	$C_{46}H_{86}NO_7P$	PC
	LMGP01090045	PC(20:3(8Z,11Z,14Z)/P-18:0)	$C_{46}H_{86}NO_7P$	PC
	LMGP01090039	PC(20:2(11Z,14Z)/P-18:1(9Z))	$C_{46}H_{86}NO_7P$	PC
	LMGP01090041	PC(O-18:0/20:4(5E,8E,11E,14E))	$C_{46}H_{86}NO_7P$	PC
	LMGP01090045	PC(O-18:0/20:4(5Z,8Z,11Z,14Z))	$C_{46}H_{86}NO_7P$	PC

Note: [1] Lipidmaps accession numbers. [2] PC (phosphatidylcholine) and PE (phosphatidylethanolamine) PL classes. Grey highlighted lines corresponds to the PL species characterized by LC-ESI-MS/MS analyses.

Table S3. LC-ESI-MS relative content determination of PLs observed during MALDI MSI analyses of MCF-7 and MDA-MB-435 tumor sections.

^{MSI} m/z_{obs} [1]	MCF-7		MDA-MB-435	
	$^{LC-MS}$ m/z_{obs} [2]	Relative content [3]	$^{LC-MS}$ m/z_{obs} [2]	Relative content [3]
744.49419[4]	-	-	-	-
744.55385[5]	744.54992	1	744.55021	0.25
744.59019[5]	744.59332	1	744.58834	18.95

Note: [1] Experimental *m/z* values acquired during MALDI MSI analyses. [2] Experimental *m/z* values acquired during LC-ESI-MS analyses. [3] Relative PL content normalized against MCF-7 PL content. [4] Adducted ($[M+K]^+$) ion specie presented in Table 3. [5] Protonated ($[M+H]^+$) ion species presented in Table 3.

Figure S5. Relative intensities of peaks of three couples low-abundant PLs from MCF-7, MDA-MB-231 and MDA-MB-435 samples. (**A**) PLs with *m/z* values comprised between *m/z* 744.45 and 744.60. (**B**) PLs with *m/z* values comprised between *m/z* 796.50 and 796.65. And (**C**) PLs with *m/z* values comprised between *m/z* 790.50 and 790.58. Arrows indicate the patterns of relative intensities directly associated with the aggressive phenotype of each tumor.

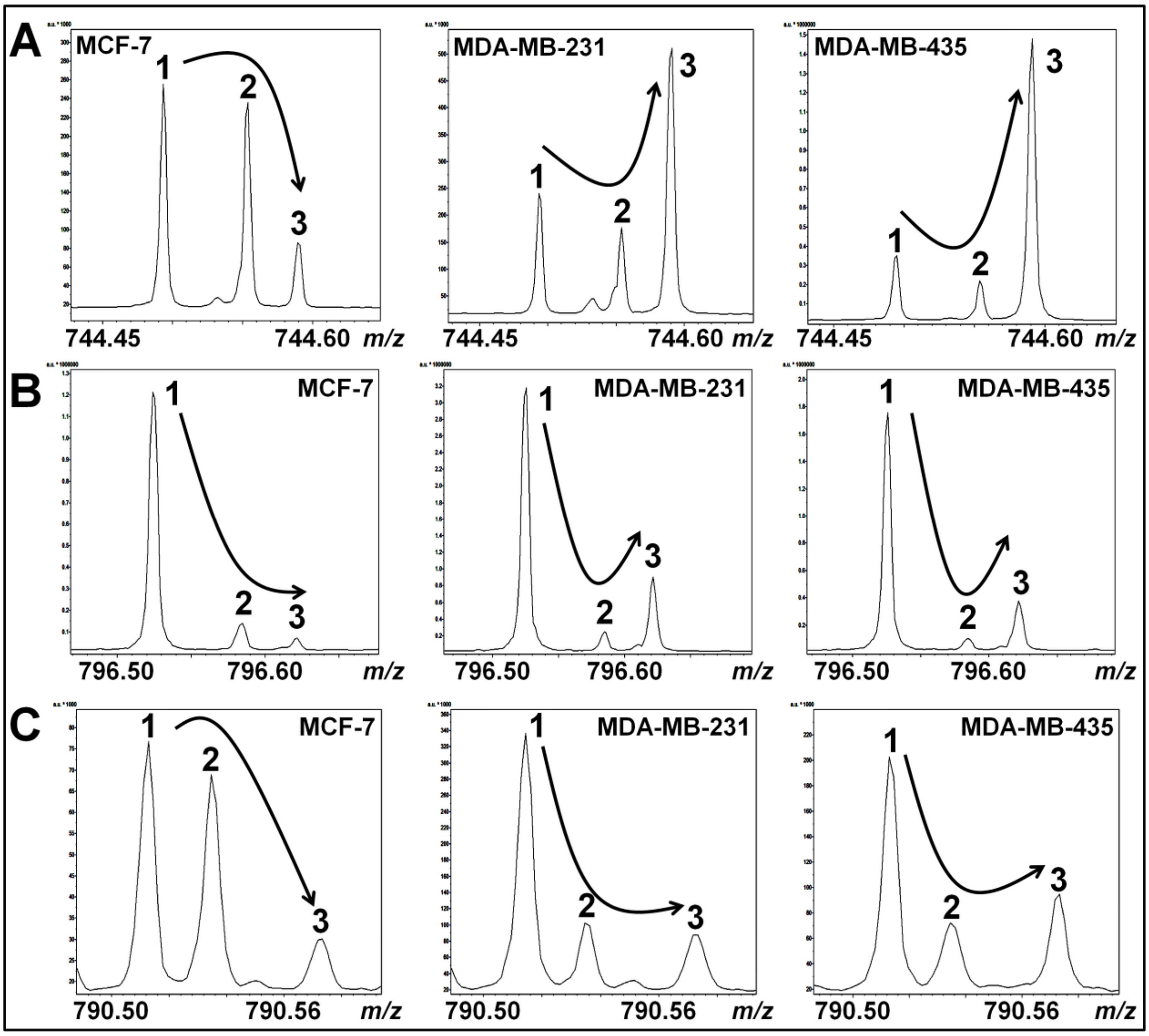

Figure S6. pLSA analysis from MALDI MSI data of MCF-7, MDA-MB-231 and MDA-MB-435 samples. Excerpts of the *m/z* range showing results of pLSA analyses of MCF-7 *versus* MDA-MB-231 (**A**) and MCF-7 *versus* MDA-MB-435 (**B**) for peaks comprised between *m/z* 744.45 and 744.60, *m/z* 796.50 and 796.65 and *m/z* 790.50 and 790.58. Bar plots result from the analysis of two components. Blue bars correspond to PL species localized in MCF-7 tumor section and red bars those ones localized in MDA-MB-231 (**A**) or MDA-MB-435 (**B**) tumor sections, respectively. At these *m/z* values, the blue and red bar plots have unequal intensity for the two component spectra, indicative of a discriminatory power from the *m/z* values. Arrows indicate the patterns of relative intensities directly associated with the aggressive phenotype of each tumor [blue for MCF-7 and red for MDA-MB-231 in (**A**) or MDA-MB-435 in (**B**)].

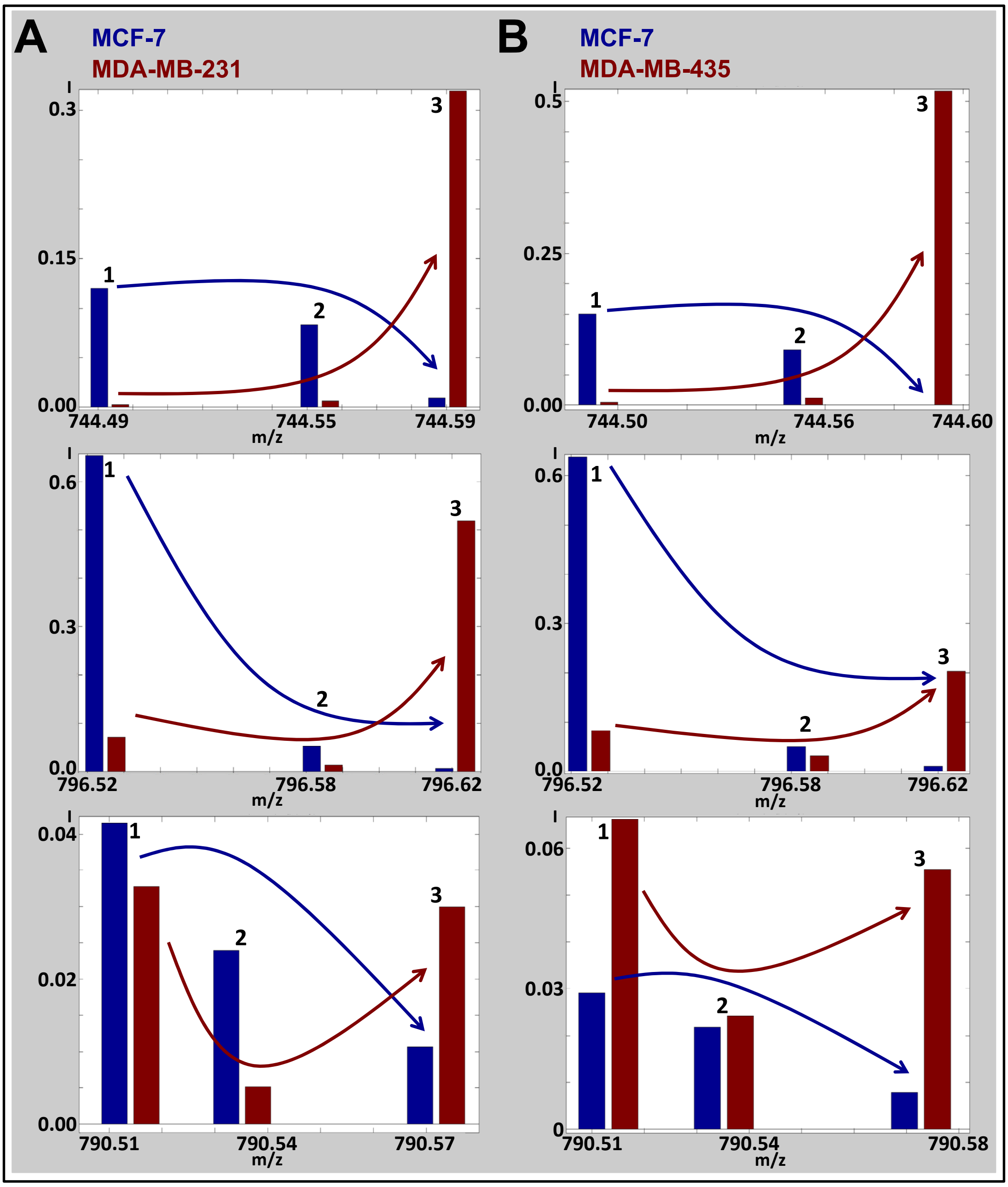

Figure S7. LC-ESI-MS analyses of lipid extracts from MCF-7 (**A**) and MDA-MB-231 (**B**) and MDA-MB-435 tissues (**C**). Upper panels correspond to the base peak chromatograms of the LC-ESI-MS analyses of lipid extracts. Lower panels correspond to XIC ranged from *m/z* values 744.5 to 744.6.

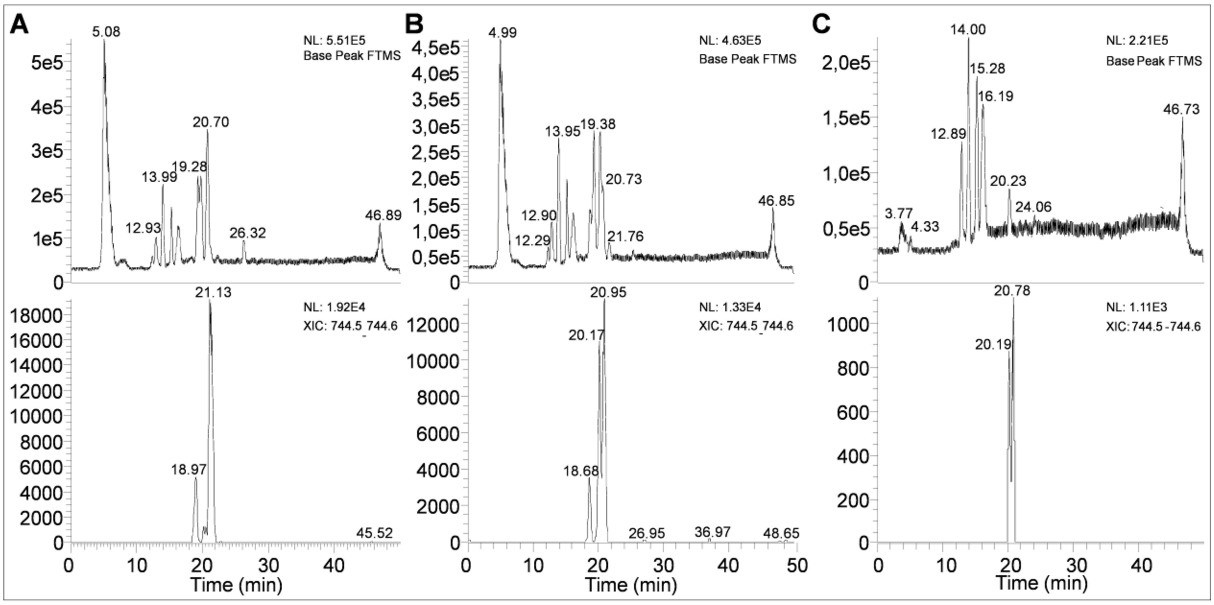

Figure S8. LC-ESI-MS analyses of lipid extracts from MCF-7 and MDA-MB-231 tissues. (**A**) Total ion currents (TIC) of LC-ESI-MS analyses. Insets show the extract ion currents ranged from *m/z* values 796 to 797. (**B**), (**C**) and (**D**) Exact mass measurements acquired with the FTICR analyzer. Insets show MS/MS spectra at respective retention times. The visualization of an ion with an *m/z* value of 184 indicates that the PL is a PC.

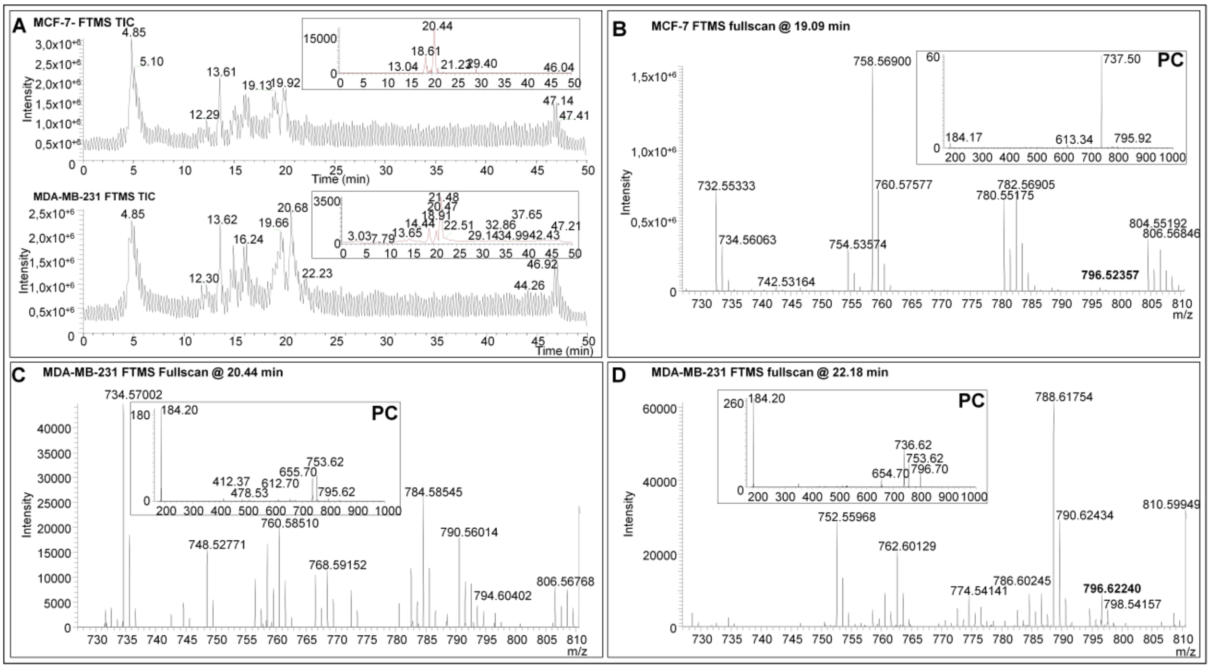

Figure S9. Specific localization of minor PL species in MDA-MB-435 tumor sections. Schema of the gradient of minor PL species from the necrotic area to the proliferating tumor region in a section of tumor induced by MDA-MB-435 cells. Dotted lines delineate necrosis "*N*" and tumor "*T*" areas.

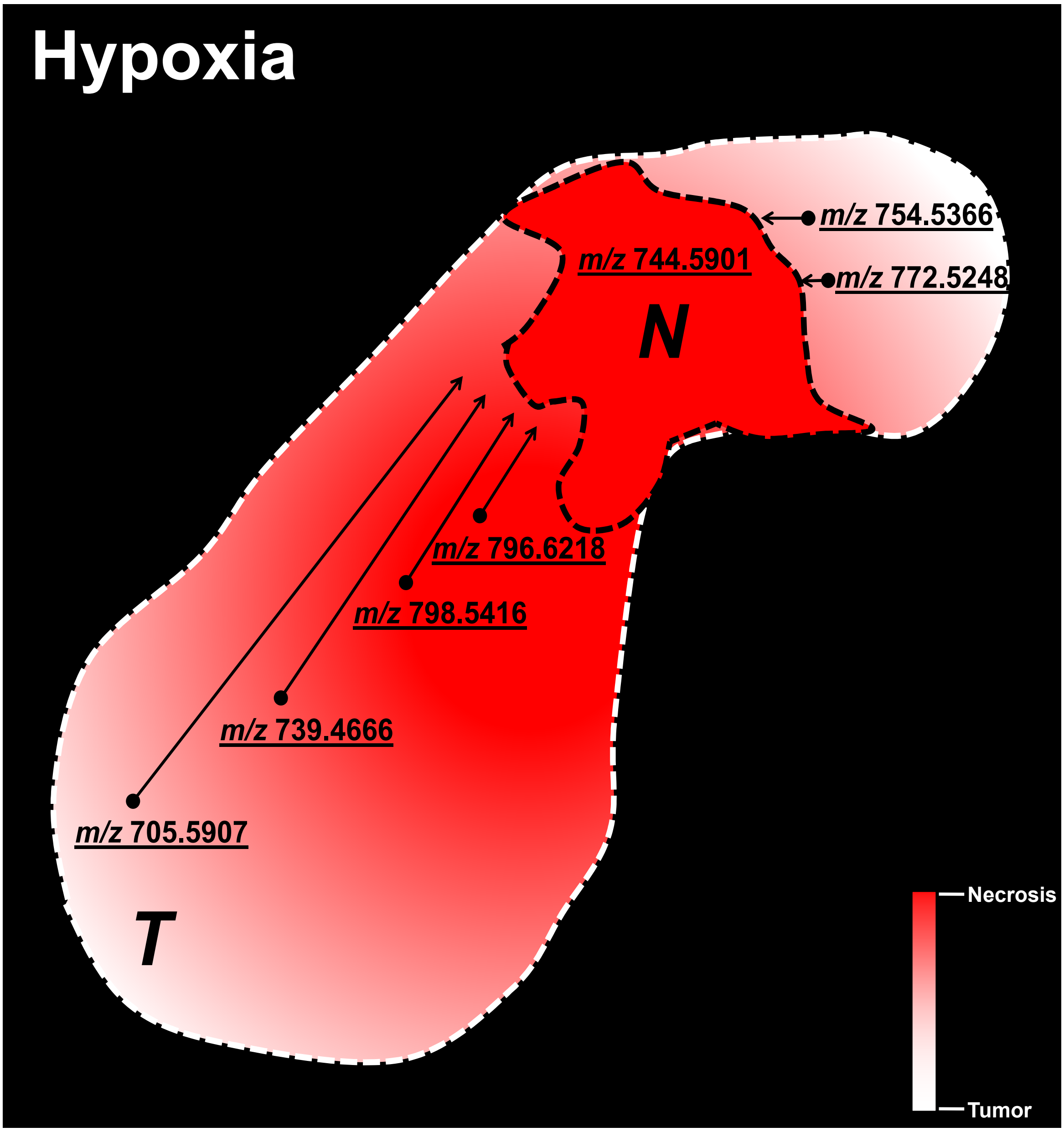

Figure S10. Localization of PC(18:0/18:2) in MCF7 and MDA-MB-435 tumor sections. MALDI MSI ion images representing the localization of PC(18:0/18:2) (*m/z* values of 786.6008) in sections of tumor induced by MCF-7 cells (**A**) and MDA-MB-435 (**B**).

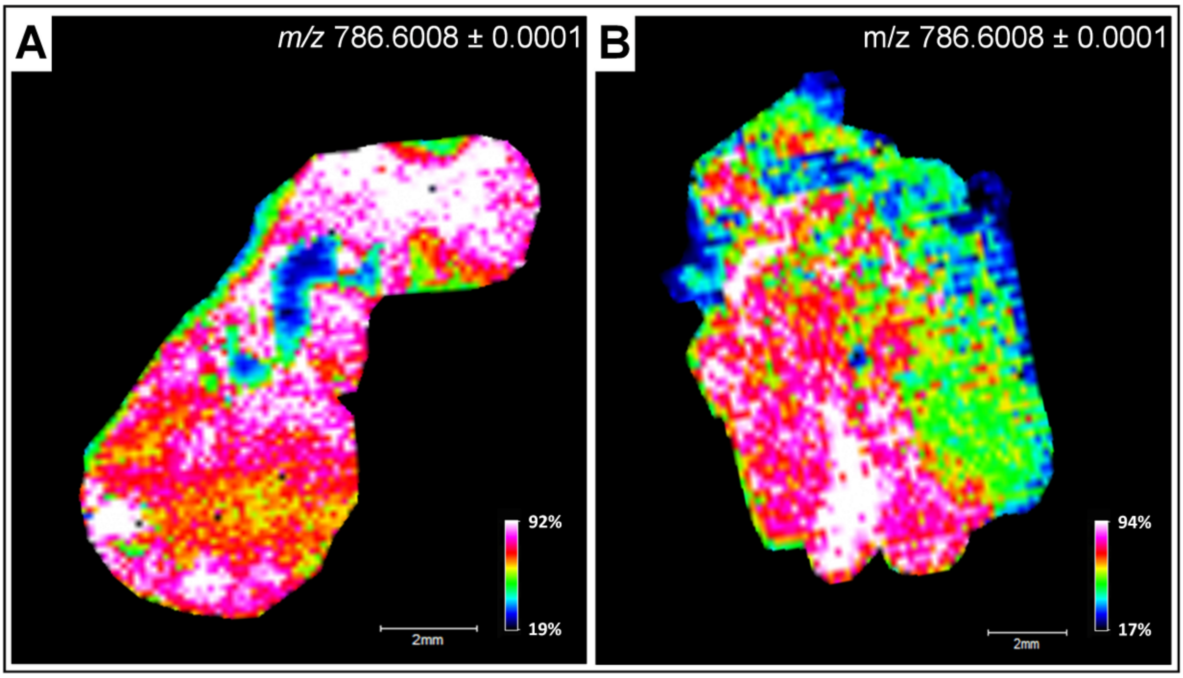

© 2013 by the authors; licensee MDPI, Basel, Switzerland. This article is an open access article distributed under the terms and conditions of the Creative Commons Attribution license (http://creativecommons.org/licenses/by/3.0/).
